# Anti‐Inflammatory Effects of *Stachys pilifera* Extracts in LPS‐Stimulated RAW264.7 Macrophages

**DOI:** 10.1155/mi/7210911

**Published:** 2026-03-26

**Authors:** Zeinab Salehpour, Mehdi Fazeli, Ehsan Barati

**Affiliations:** ^1^ Medicinal Plants Research Center, Yasuj University of Medical Sciences, Yasuj, Iran, yums.ac.ir; ^2^ Department of Basic Sciences, School of Veterinary Medicine, Shiraz University, Shiraz, Iran, shirazu.ac.ir; ^3^ Department of Chemistry, Howard University, Washington, DC 20059, USA, howard.edu

**Keywords:** anti-inflammatory, cyclooxygenase-2 (COX-2), nitric oxide (NO), nuclear factor-κB (NF-κB), prostaglandin E2 (PGE_2_), *Stachys pilifera* Benth

## Abstract

**Objective:**

Inflammation is a fundamental biological response that serves to protect the body from physical damage and harmful stimuli. However, chronic inflammation is implicated in the pathogenesis of various inflammatory diseases. *Stachys pilifera* Benth. has been traditionally used for its anti‐inflammatory properties, yet its precise mechanisms of action remain to be fully elucidated. This study aims to investigate the effects of *S. pilifera* Benth. extract and its fractions on lipopolysaccharide (LPS)‐induced inflammatory responses in RAW264.7 macrophage cells.

**Methodology:**

The anti‐inflammatory potential of the methanolic extract and its ethyl acetate, butanol, and water fractions were evaluated by assessing their inhibitory effects on nitric oxide (NO) and prostaglandin E2 (PGE_2_) production. Additionally, nuclear factor‐κB (NF‐κB) concentration and cyclooxygenase‐2 (*COX-2*) gene expression were analyzed in LPS‐stimulated macrophage cells.

**Results:**

The methanolic extract, ethyl acetate fraction, and butanol fraction significantly reduced the production of NO and PGE_2_, decreased NF‐κB concentration, and suppressed *COX-2* mRNA expression in a dose‐dependent manner. The butanol fraction exhibited the most potent inhibition of NO production (IC_50_ = 37.38 ± 10.27 µg/mL), whereas the ethyl acetate fraction was the strongest suppression of PGE_2_ synthesis (IC_50_ = 86.32 ± 5.51 µg/mL). In contrast, the water fraction did not have a significant effect on these inflammatory markers.

**Conclusion:**

By downregulating NF‐κB activity, *S. pilifera* Benth. effectively modulates the expression of key proinflammatory mediators, including inducible NO synthase (iNOS) and *COX-2*, ultimately leading to reduced NO and PGE_2_ production. These findings suggest that *S. pilifera* Benth. may exert its anti‐inflammatory effects through inhibition of the NF‐κB signaling pathway, offering potential therapeutic applications in the management of chronic inflammatory diseases.

## 1. Introduction

Inflammation is a fundamental biological response designed to protect the body from physical damage and harmful stimuli [1–3]. It is generally classified into two types: acute and chronic. Acute inflammation provides a rapid response to pathogenic organisms, whereas chronic inflammation results from the prolonged activation of the immune system, which can contribute to pathological conditions. In both types of inflammation, mononuclear cells, such as macrophages and lymphocytes, play a crucial role [4].

Macrophages are key immune cells activated in response to inflammatory stimuli, serving a vital function in defending the body against harmful agents [5–10]. Upon activation, macrophages produce a variety of inflammatory mediators, including interleukin‐1β (IL‐1β), tumor necrosis factor‐α (TNF‐α), nitric oxide (NO), and prostaglandins (PGs) [11].

Lipopolysaccharide (LPS), a major component of the outer membrane of Gram‐negative bacteria, is a potent stimulator of macrophages, triggering the release of inflammatory cytokines such as TNF‐α and ILs, along with proinflammatory mediators like NO and PG E2 (PGE_2_) [12, 13].

PGE_2_, a key inflammatory mediator, is synthesized from arachidonic acid through the action of cyclooxygenase‐2 (COX‐2) [14]. PGs play a central role in mediating inflammatory responses, including pain, fever, and tissue swelling [15]. NO, a reactive free radical, is generated from L‐arginine by constitutive NO synthase (cNOS) and inducible NOS (iNOS) in mammalian cells and is widely released during inflammatory conditions [16, 17]. Studies have demonstrated that LPS‐induced inflammation is mediated through intracellular signaling pathways, particularly nuclear factor‐κB (NF‐κB) and mitogen‐activated protein kinase (MAPK) pathways [18]. Consequently, LPS‐stimulated macrophages are widely used as an in vitro model for studying inflammatory processes and evaluating potential therapeutic agents for inflammatory diseases [19].

NF‐κB is a pivotal regulator of immune and inflammatory responses, modulating the expression of iNOS, COX‐2, and various proinflammatory cytokines [20]. Given its role in inflammatory pathogenesis, NF‐κB is a key therapeutic target in the search for novel anti‐inflammatory compounds [21]. Although inflammation is an essential immune response, excessive or dysregulated inflammatory activity can contribute to a wide range of diseases, including septic shock, diabetes, arthritis, and cancer [22, 23]. Current anti‐inflammatory treatments, including corticosteroids and nonsteroidal anti‐inflammatory drugs (NSAIDs) such as dexamethasone, aspirin, and indomethacin, are widely used to regulate inflammatory responses. However, these drugs are often associated with significant side effects, necessitating the search for safer and more effective alternatives [24–26].

The *Stachys* genus, belonging to the Lamiaceae family, comprises ~270 species, predominantly distributed across tropical and subtropical regions. Among these, 34 species have been identified in Iran, with 13 being endemic [27, 28].

Phytochemical investigations of *Stachys* species have revealed the presence of flavonoids, phenylethanoid glycosides, diterpenes, saponins, terpenoids, and steroids [29–31]. Furthermore, various *Stachys* species have been reported to exhibit antibacterial, anti‐inflammatory, hepatoprotective, and antioxidant properties [32–35].

*S. pilifera* Benth., an endemic species of Iran, is traditionally used in herbal medicine for the treatment of infections, asthma, rheumatism, and other inflammatory disorders [28]. Previous studies have reported its antiviral and antitumor activities, particularly from its *n*‐butanolic extract [36]. Additionally, animal model studies have demonstrated the anti‐inflammatory effects of its hydroalcoholic aerosol extract [37].

In recent years, drug development strategies have increasingly emphasized both efficacy and safety. Ethnopharmacology, which integrates traditional knowledge with modern drug discovery, has emerged as a promising approach to identifying novel therapeutic agents with minimal side effects.

## 2. Materials and Methods

### 2.1. Chemicals and Reagents

LPS; Sigma–Aldrich, St. Louis, MO, USA, Dulbecco’s Modified Eagle’s Medium (DMEM; Gibco BRL, Life Technologies, Paisley, Scotland), fetal bovine serum (FBS; Gibco BRL, Life Technologies, Paisley, Scotland), penicillin (Gibco BRL, Life Technologies, Paisley, Scotland; 100 IU/mL), streptomycin (Gibco BRL, Life Technologies, Paisley, Scotland; 100 μg/mL), trypsin‐EDTA (Gibco BRL, Life Technologies, Paisley, Scotland), 3‐(4,5‐Dimethylthiazol‐2‐yl)‐2,5‐diphenyl tetrazolium bromide (MTT; Sigma–Aldrich Corp, St. Louis, MO, USA), NF‐κB p65 ELISA Kit (Abcam, Cambridge, MA, USA), PGE_2_ Parameter Assay Kit (R&D Systems, Minneapolis, MN, USA), GeneAll RiboEx Total RNA Extraction Kit (GeneAll Biotechnology, Seoul, Korea), PrimeScript RT Reagent Kit (Takara Bio Inc., Tokyo, Japan), TB Green Premix Ex Taq II (Takara Bio Inc., Tokyo, Japan), methanol, *n*‐hexane, chloroform, ethyl acetate, *n*‐butanol (BuOH) (analytical grade; Merck, Darmstadt, Germany), distilled water (laboratory grade).

### 2.2. Preparation of Plant Extract and Its Fractions

The aerial parts of the plant material were shade‐dried at ambient temperature (25–30°C) and finely powdered.

### 2.3. Maceration–Percolation Extraction

A standardized maceration–percolation protocol was employed in accordance with the United States Pharmacopeia (USP) <561> guidelines to ensure reproducibility [38]. Briefly, 1 kg of powdered material was initially macerated in 10 L of 80% methanol (1:10 w/v) for 24 h at 25–30°C with occasional agitation. Percolation was subsequently carried out in a conical percolator at a flow rate of 4–6 drops per minute until complete solvent exhaustion. The extraction cycle was repeated three times with fresh solvent to maximize recovery of polar metabolites (>90% yield), and all percolates were pooled and concentrated under reduced pressure using a rotary evaporator at 35°C.

### 2.4. Solvent Partitioning and Fractionation

**Figure 1:**
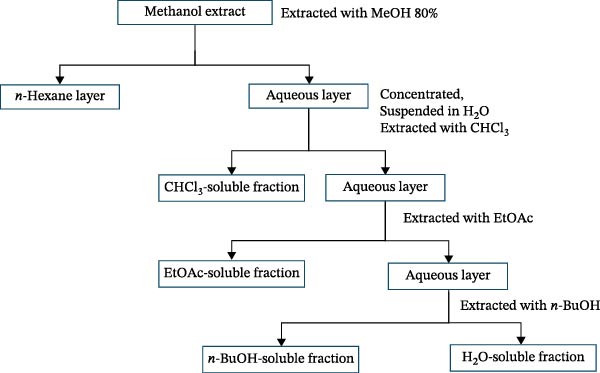
Steps of extraction and fractionation of *Stachys pilifera* Benth.

The crude methanolic extract was reconstituted in distilled water (1:10 w/v) and defatted three times with *n*‐hexane (1:1 v/v) using a separatory funnel, with each cycle involving 15 min of gentle shaking. The resulting aqueous phase was subjected to sequential liquid–liquid partitioning following the Kupchan scheme: chloroform (3 × 1:1 v/v), ethyl acetate (3 × 1:1 v/v), and BuOH (3 × 1:1 v/v). Each phase was allowed to equilibrate for 30 min before separation. The residual aqueous layer was collected as the water fraction (Figure 1). All fractions were concentrated under reduced pressure at 35°C. Fractions were stored at 4°C in airtight, desiccated containers. This polarity‐based fractionation approach is consistent with established pharmacognostic methods for enrichment of bioactive constituents in early‐stage pharmacological screening [39, 40].

### 2.5. Cell Culture

The RAW264.7 macrophage cell line was obtained from the Cell Bank of the Pasteur Institute of Iran. Cells were cultured in DMEM supplemented with 10% FBS and 1% streptomycin/penicillin. Cultures were maintained at 37°C in a humidified incubator with 5% CO_2_.

### 2.6. Cell Cytotoxicity Assay

The cytotoxic effects of *S. pilifera* Benth. extracts were assessed in LPS‐stimulated RAW264.7 macrophages using the MTT assay. Cells (1 × 10^4^ per well) were seeded into 96‐well plates and incubated for 24 h. Following incubation, cells were treated with varying concentrations (25, 50, 100, 200, and 400 μg/mL) of the methanolic extract and its fractions in the presence of LPS (1 μg/mL) for 24 h. MTT reagent was added to each well, and cells were incubated for an additional 4 h at 37°C. The medium was then replaced with dimethyl sulfoxide (DMSO), and incubation continued for 15 min in darkness. Absorbance was measured at 570 nm using a microplate reader. Cytotoxicity was expressed as a percentage using the following formula:

Percentage of toxicity= Absorbance of control−absorbance of sample/absorbance of control×100.

Since the methanolic extract and all fractions exhibited high cytotoxicity at 200 and 400 μg/mL, the lower concentrations (25, 50, and 100 μg/mL), which were noncytotoxic, were selected for further experiments, while the highly toxic chloroform fraction was excluded.

### 2.7. NO Measurement

RAW264.7 macrophages (1 × 10^4^ cells/well) were seeded into 96‐well microplates and incubated for 24 h. Cells were then treated with 25, 50, and 100 μg/mL of methanolic extract, ethyl acetate, butanol, and water fractions. After 1 h, cells were stimulated with LPS (1 μg/mL) and incubated for an additional 24 h. The concentration of NO in the culture medium was determined using the Griess reaction [41]. Briefly, 100 μL of the cell culture supernatant was mixed with an equal volume of Griess reagent in a 96‐well plate, and absorbance was measured at 550 nm after 10 min. NO concentration was calculated using a sodium nitrite (NaNO_2_) standard curve.

### 2.8. PGE_2_ Measurement

RAW264.7 cells (1 × 10^4^ cells/well) were seeded into 96‐well plates, incubated for 24 h, and treated with 25, 50, and 100 μg/mL of the methanolic extract, ethyl acetate, butanol, and water fractions. After 1 h, cells were stimulated with LPS (1 μg/mL) and incubated for 24 h. The concentration of PGE_2_ in the culture medium was measured using an ELISA kit (R&D Systems, Minneapolis, MN, USA) according to the manufacturer’s instructions. Absorbance was recorded at 405 nm.

### 2.9. NF‐κB Inflammatory Factor Measurement

RAW264.7 macrophages (1 × 10^4^ cells/well) were cultured overnight in 96‐well microplates. Cells were then treated with 25, 50, and 100 μg/mL of the methanolic extract, ethyl acetate, butanol, and water fractions in the presence of LPS (1 μg/mL) for 24 h. NF‐κB p65 ELISA was performed using nuclear extracts according to the manufacturer’s protocol (Abcam, USA) with absorbance measured at 450 nm.

### 2.10. Expression of *COX-2* Gene

RAW264.7 cells (1 × 10^7^ cells/well) were seeded into six‐well plates and incubated for 24 h. Cells were then treated with 25, 50, and 100 μg/mL of the methanolic extract, ethyl acetate, butanol, and water fractions in the presence of LPS (1 μg/mL) for 24 h. Cells were harvested using trypsin, and total RNA was extracted using the GeneAll RiboEx kit (GeneAll, Korea) according to the manufacturer’s protocol. Complementary DNA (cDNA) was synthesized using the cDNA synthesis kit (Takara, Tokyo, Japan).

The sequences of specific primers for *COX-2* and *HPRT1* (internal reference gene) are shown in Table 1. Gene expression was analyzed using the Takara CyberGrin kit and real‐time PCR (Bio‐Rad, CFX96, USA). The specificity of the amplified product was confirmed using melting curve analysis. Relative gene expression levels were calculated using the 2^−ΔΔCt^ method. Each experiment included three biological replicates, with three technical replicates per sample.

**Table 1:** Primer sequences used for real‐time PCR analysis.

Gene	Oligonucleotides	Prime type (forward/reverse)
*HPRT1*	5′‐GCCCTGGCGTCGTGATTAG‐3′ 5′‐TCGAGCAAGACGTTCAGTCC‐3	F1 R1

*COX-2*	5′‐CCTCAGCTCCACAGCCAGACG‐3′ 5′‐CTCGGTTTTGACATGGGTGGG‐3′	F1 R1

*Note:* The table presents the specific primer sequences utilized for real‐time PCR analysis in this study. The primers were designed to amplify target genes involved in inflammatory response, including *COX-2*, with *HPRT1* serving as the internal reference gene. These primers were selected based on previous studies and optimized for efficiency and specificity in gene expression analysis.

### 2.11. PCR Conditions

Real‐time PCR cycling conditions were as follows:

• Initial denaturation: 94°C for 10–15 s
• Amplification: 45 cycles of
  • Denaturation: 94°C for 30 s
  • Annealing: 58°C for 30 s
  • Extension: 72°C for 1 min

### 2.12. Statistical Analysis

Data were presented as the mean ± standard deviation (SD) of at least three independent experiments. Statistical analyses were performed using one‐way analysis of variance (ANOVA), followed by post hoc tests where applicable. Differences were considered statistically significant at *p*  < 0.05. Statistical analysis was conducted using SPSS software (version 23 for Windows).

## 3. Results

### 3.1. Cytotoxic Effects of *S. pilifera* Benth. Extracts on RAW264.7 Cell

**Figure 2:**
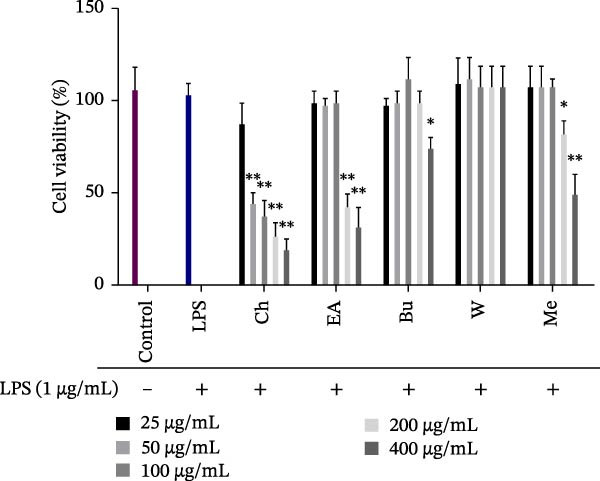
Cytotoxicity of *Stachys pilifera* Benth. methanolic extract and its fractions in LPS‐stimulated RAW264.7 cells. RAW264.7 macrophages were treated with various concentrations (25, 50, 100, 200, and 400 µg/mL) of *Stachys pilifera* Benth. methanolic extract and its fractions in the presence of LPS (1 µg/mL). Cytotoxicity was assessed using the MTT assay. Data are presented as the mean ± SD of triplicate measurements (*n* = 3) from three independent experiments. *p*  < 0.05 ( ^∗^) and *p*  < 0.001 ( ^∗∗^) indicate statistically significant differences compared to the LPS‐treated group. Abbreviations: Bu, butanol fraction; Ch, chloroform fraction; EA, ethyl acetate fraction; Me, methanolic extract; W, water fraction.

The cytotoxicity of the methanolic extract and its ethyl acetate, butanol, and water fractions in LPS‐stimulated RAW264.7 macrophages was evaluated using the MTT assay. The chloroform fraction exhibited high cytotoxicity at low concentrations and was therefore excluded from further experiments. The methanolic extract and the remaining fractions were nontoxic to RAW264.7 cells at concentrations up to 100 μg/mL (Figure 2). Based on these findings, concentrations of 25, 50, and 100 μg/mL were selected for subsequent experiments.

### 3.2. Effect of *S. pilifera* Benth. Extracts on NO Production in LPS‐Stimulated RAW264.7 Cells

**Figure 3:**
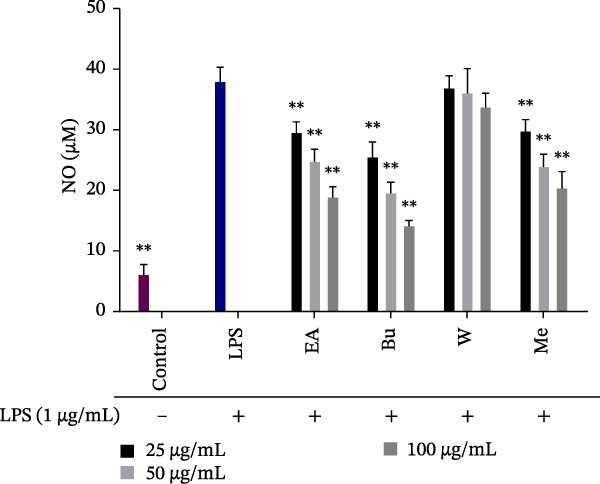
Inhibitory Effects of *Stachys pilifera* Benth. methanolic extract and its fractions on NO production in LPS‐stimulated RAW264.7 cells. RAW264.7 macrophages were treated with *Stachys pilifera* Benth. methanolic extract and its fractions at concentrations of 25, 50, and 100 µg/mL, followed by stimulation with LPS (1 µg/mL) for 24 h. The concentration of nitric oxide (NO) in the culture medium was quantified using the Griess assay. Data are presented as the mean ± SD of triplicate measurements (*n* = 3) from three independent experiments. *p*  < 0.05 ( ^∗^) and *p*  < 0.001 ( ^∗∗^) indicate statistically significant differences compared to the LPS‐treated group.

LPS stimulation significantly increased NO levels in the LPS‐treated group compared with the controls group (*p* < 0.001). The methanolic extract, ethyl acetate fraction, and butanol fraction significantly reduced NO production in a dose‐dependent manner (*p* < 0.001), whereas the water fraction had no significant effect at any concentration (Figure 3).

### 3.3. Effect of *S. pilifera* Benth. Extracts on PGE_2_ Production in LPS‐Stimulated RAW264.7 Cells

**Figure 4:**
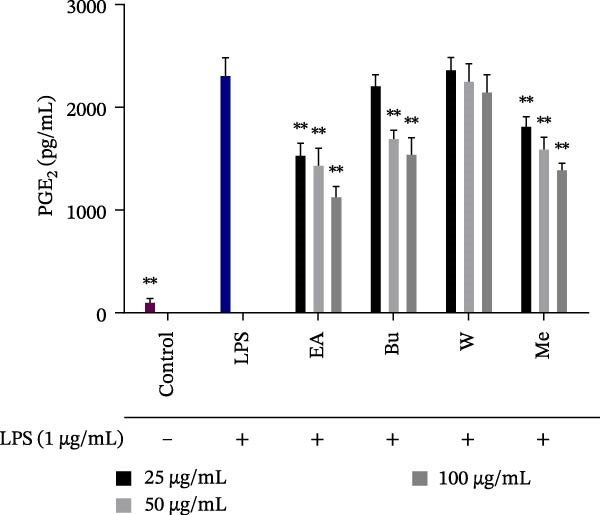
Effect of *Stachys pilifera* Benth. methanolic extract and its fractions on prostaglandin E2 (PGE_2_) production in LPS‐stimulated RAW264.7 cells. RAW264.7 macrophages were treated with *Stachys pilifera* Benth. methanolic extract and its fractions at concentrations of 25, 50, and 100 µg/mL, followed by stimulation with LPS (1 µg/mL). The concentration of prostaglandin E2 (PGE_2_) in the culture medium was measured using an ELISA assay. Data are presented as the mean ± SD of triplicate measurements (*n* = 3) from three independent experiments. *p*  < 0.05 ( ^∗^) and *p*  < 0.001 ( ^∗∗^) indicate statistically significant differences compared to the LPS‐treated group.

PGE_2_ levels were markedly elevated in LPS‐treated cells (*p* < 0.001). The methanolic extract and ethyl acetate fractions significantly suppressed PGE_2_ at all tested concentrations (*p*  < 0.001). The butanol fraction showed significant inhibition at 50 and 100 µg/mL (*p* < 0.001) but not at 25 µg/mL, while the aqueous fraction remained ineffective (Figure 4).

### 3.4. Effect of *S. pilifera* Benth. Extracts on NF‐κB (p65) Concentration in LPS‐Stimulated RAW264.7 Cells

**Figure 5:**
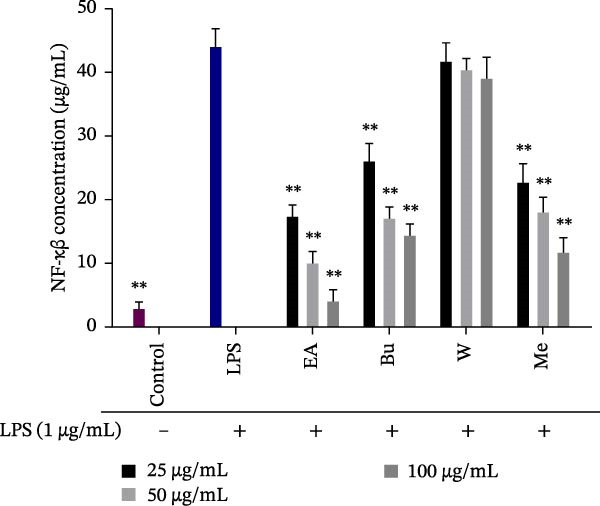
Inhibitory effect of *Stachys pilifera* Benth. methanolic extract and its fractions on NF‐κB (p65) concentration in LPS‐stimulated RAW264.7 cells. RAW264.7 macrophages were pretreated with *Stachys pilifera* Benth. methanolic extract and its fractions at concentrations of 25, 50, and 100 µg/mL for 1 h before stimulation with LPS (1 µg/mL). NF‐κB (p65) concentration was measured to assess the inhibitory effect of the extracts. Data are presented as the mean ± SD of triplicate measurements from three independent experiments. *p*  < 0.05 ( ^∗^) and *p*  < 0.001 ( ^∗∗^) indicate statistically significant differences compared to the LPS‐treated group. Abbreviations: Bu, butanol fraction; EA, ethyl acetate fraction; Me, methanolic extract; W, water fraction.

LPS stimulation significantly increased intracellular NF‐κB levels (*p* < 0.001). The methanolic extract, ethyl acetate fraction, and butanol fraction significantly decreased NF‐κB levels in a dose‐dependent manner (*p* < 0.001), while the water fraction did not affect NF‐κB concentration (Figure 5).

### 3.5. Effect of *S. pilifera* Benth. Extracts on *COX-2* Gene Expression in LPS‐Stimulated RAW264.7 Cells

**Figure 6:**
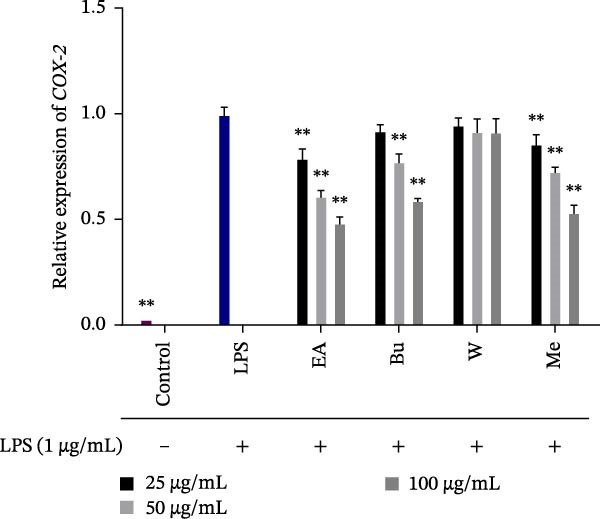
Effect of *Stachys pilifera* Benth. methanolic extract and its fractions on *COX-2* expression in LPS‐stimulated RAW264.7 macrophages. RAW264.7 macrophages were pretreated with *Stachys pilifera* Benth. methanolic extract and its fractions at concentrations of 25, 50, and 100 µg/mL for 1 h before stimulation with LPS (1 µg/mL). Cells were incubated for 24 h, after which RNA was extracted, and relative *COX-2* expression was quantified using real‐time PCR, with *HPRT1* as the reference gene. Data are presented as the mean ± SD of three independent experiments. *p*  < 0.05 ( ^∗^) and *p*  < 0.001 ( ^∗∗^) indicate statistically significant differences compared to the LPS‐treated group.

*COX-2* expression was significantly upregulated following LPS stimulation (*p* < 0.001). The methanolic extract, ethyl acetate fraction, and butanol fraction significantly reduced *COX-2* expression in a dose‐dependent manner (*p* < 0.001), whereas the water fraction at all concentrations and the butanol fraction at 25 μg/mL did not show a significant effect compared to LPS‐treated cells (Figure 6).

### 3.6. Quantitative IC_50_ Analysis

Quantitative analysis of anti‐inflammatory activity revealed a marked fraction‐dependent selectivity (Table 2). Among the tested fractions, the BuOH fraction exhibited the most potent inhibition of NO production (IC_50_ = 37.38 ± 10.27 µg/mL), whereas the ethyl acetate (EtOAc) fraction was the strongest suppression of PGE_2_ synthesis (IC_50_ = 86.32 ± 5.51 µg/mL). The methanolic extract displayed intermediate activity for both mediators (IC_50_ = 74.27 ± 8.59 and 165.1 ± 5.56 µg/mL for NO and PGE_2_, respectively), while the aqueous fraction showed minimal efficacy (IC_50_ > 200 µg/mL).

**Table 2:** IC_50_ value for inhibition of NO and PGE_2_ production by *Stachys pilifera* Benth.

Sample	IC_50_ (NO inhibition, µg/mL)	IC_50_ (PGE_2_ inhibition, µg/mL)
Ethyl acetate fraction (EtOAc)	68.41 ± 9.65	86.32 ± 5.51
*n*‐butanol fraction (BuOH)	37.38 ± 10.27	157.0 ± 9.13
Water fraction (W)	> 200	> 200
Methanolic extract (ME)	74.27 ± 8.59	165.1 ± 5.56

*Note:* Methanolic extract and its fractions. Data are presented as mean ± SD of three independent experiments.

## 4. Discussion

Macrophages play a central role in immune defense, particularly in the inflammatory response. Upon exposure to stimulatory factors such as LPS, macrophages activate inflammatory pathways and release proinflammatory mediators, including TNF‐α, IL‐1β, NO, PGE_2_, and *COX-2* [42–44]. LPS, a component of the Gram‐negative bacterial membrane, is recognized by the Toll‐like receptor 4 (TLR4), which subsequently activates the TLR4‐NF‐κB signaling pathway, leading to an inflammatory response [45].

The inflammatory response in RAW264.7 macrophages that are stimulated by LPS leads to the release of a large number of inflammatory cytokines (TNF‐α, IL‐1β, NO, and IL‐6) and inflammatory mediators (PGE_2_, COX‐2, and iNOS) [46]. NO is a free radical with many physiological and pathological functions, which is produced by oxidation of L‐arginine amino acid by NOS enzyme.

NF‐κB is a key transcription factor regulating inflammatory mediators and cytokines. Under normal conditions, NF‐κB is sequestered in the cytoplasm by the inhibitory protein IκB. Upon activation by external stimuli, IκB is phosphorylated, leading to NF‐κB translocation into the nucleus, where it upregulates genes encoding proinflammatory cytokines and enzymes such as *COX-2* and iNOS [20, 47, 48]. Targeting NF‐κB activation is a well‐established strategy for controlling inflammation [49]. The results of this study indicate that *S. pilifera* Benth. extracts significantly inhibit NF‐κB activity, suggesting their potential for therapeutic use in inflammatory disorders.

Flavonoids, widely recognized for their anti‐inflammatory properties, exert their effects by modulating inflammatory enzymes, reducing oxidative stress, and inhibiting transcription factors such as NF‐κB and AP‐1 [50–53]. Previous studies have demonstrated that plant polyphenols exert anti‐inflammatory effects in LPS‐stimulated macrophages by inhibiting *COX-2*, iNOS, and NF‐κB activity [54–58]. Specifically, Zarezade et al. [59] demonstrated that the hydroalcoholic extract of *S*. *pilifera* Benth. contains a high level of bioactive compounds, with total flavonoid content of 660.79 ± 10.06 mg rutin equivalents (RE)/g extract and total phenolic content of 101.35 ± 2.96 mg gallic acid equivalents (GAE)/g extract. These results support the phytochemical richness of *S. pilifera* and are consistent with the biological activity observed in our study [59].The findings of the present study align with these reports, indicating that *S. pilifera* Benth., which is rich in phenolic compounds and flavonoids, may exert its anti‐inflammatory effects through the suppression of NF‐κB signaling.

The *S. pilifera* Benth. fractions were prepared using a standard polarity‐based fractionation strategy, widely used to separate phytochemicals according to polarity. In this approach, compounds are partitioned sequentially from moderately nonpolar to highly polar solvents (chloroform → ethyl acetate → BuOH → water) [60, 61]. This sequential partitioning produces fractions with distinct chemical profiles and differential bioactivity, facilitating targeted investigation of anti‐inflammatory and antioxidant effects [62].

The findings of this study demonstrate that *S. pilifera* Benth. extracts, particularly the methanolic extract, ethyl acetate fraction, and butanol fraction, significantly reduce NO production, PGE_2_ production, and downregulate *COX-2* expression in LPS‐stimulated macrophages. In contrast, the water fraction did not have a significant effect on these inflammatory markers. The lack of significant activity in the aqueous fraction likely reflects the poor partitioning of medium‐polarity bioactive compounds into the water phase. Many plant‐derived bioactives exhibit hydrophobic or amphiphilic properties that limit their solubility and extraction efficiency in aqueous solvents [63–65]. Consistent with this, increasing water content in extraction solvents has been shown to reduce total phenolic content and antioxidant activity, as water preferentially extracts nonphenolic constituents or less active phenolics [66]. Therefore, water, as a highly polar solvent, extracts mainly hydrophilic components such as polysaccharides and simple sugars, which lack potency against these lipid‐mediated inflammatory markers. Organic fractions yield synergistic mixtures that inhibit iNOS/COX‐2 enzymes and NF‐κB translocation more robustly. In contrast, the water fraction did not have a significant effect on these inflammatory markers.

The results from the anti‐inflammatory assays revealed that the BuOH fraction exhibited superior inhibition of NO production (IC_50_ = 37.38 ± 10.27 µg/mL) (Figure 3), whereas the EtOAc fraction demonstrated greater suppression of PGE_2_ synthesis (IC_50_ = 86.32 ± 5.51 µg/mL), (Figure 4). Differential selectivity for iNOS versus COX‐2 inhibition across plant fractions can be explained through multiple complementary mechanisms that operate independently of shared NF‐κB transcriptional regulation. Evidence suggests that selectivity may arise from direct enzyme inhibition at distinct active sites, posttranscriptional modulation, and structure‐dependent binding interactions [67–69].

Specifically, polar glycosylated flavonoids, which are enriched in the BuOH fraction, exhibit higher affinity for iNOS, leading to pronounced NO inhibition. In contrast, lipophilic aglycone compounds, predominantly present in the EtOAc fraction, preferentially interact with the NSAID‐binding site of COX‐2, resulting in enhanced PGE_2_ suppression [69]. These patterns align with fraction polarity and the chemical composition of the respective extracts [70]. Recent molecular docking and mechanistic studies further support this distinction, demonstrating that structurally related flavonols can display divergent affinities for iNOS versus COX‐2 depending on polarity, substitution pattern, and glycosylation state [69]. Analogous fraction‐dependent selectivity has also been observed in other plant‐derived systems, where enrichment of specific compound classes led to preferential inhibition of either COX‐2 activity or NO production [68]. Collectively, these observations indicate that enzyme activity and protein stability can be modulated independently of transcriptional regulation, enabling bioactive compounds to exert differential effects on iNOS and COX‐2, even when both enzymes are downstream of common upstream regulators such as NF‐κB.

This study demonstrates that *S. pilifera* Benth. exerts a pronounced anti‐inflammatory effect through inhibition of the NF‐κB signaling cascade, leading to the coordinated downregulation of key proinflammatory mediators, including iNOS and COX‐2. The consequent reduction in NO and PGE_2_ production provides a plausible mechanistic basis for the attenuation of inflammatory responses observed in vitro, consistent with previously reported findings [71].

Collectively, these findings underscore the potential relevance of *S. pilifera* Benth. in the context of chronic inflammatory pathologies, such as rheumatoid arthritis and fibrotic disorders, and are congruent with its longstanding use in traditional medicine. Importantly, the mechanistic insights reported here may inform the rational development of pathway‐targeted phytotherapeutic agents. Nonetheless, the present data are confined to in vitro models and cannot be extrapolated to in vivo efficacy or safety. Comprehensive preclinical evaluation is therefore imperative prior to any therapeutic consideration, including standard mutagenicity assessment (Ames test), repeated‐dose toxicity studies in accordance with OECD guideline 407, and evaluation of cytochrome P450 interactions, consistent with FDA and EMA regulatory frameworks for botanical products. Accordingly, rigorous in vivo efficacy and safety studies are required to substantiate the translational and therapeutic potential of this botanical candidate.

### 4.1. Limitations of the Study

While the MEOH extract and its EtOAc and BuOH fractions exhibited significant anti‐inflammatory activity, several limitations merit consideration. The 24 h endpoint captures cumulative inhibition but does not reflect the early, transient activation of NF‐κB preceding iNOS and *COX-2* expression. Future time‐course studies (0.5–24 h) with p65 Western blot, qRT‐PCR, and ELISA, complemented by nuclear/cytoplasmic fractionation or immunofluorescence, are needed to clarify kinetics and nuclear translocation.

Direct quantification of iNOS mRNA and protein is necessary to fully characterize pathway specificity. Expanding experiments to additional macrophage lines, primary macrophages, and in vivo chronic inflammation models will improve translational relevance. Comprehensive HPLC‐MS/MS profiling and fraction‐specific phenolic/flavonoid analyses will enable structure–activity correlations. Finally, systematic evaluation of NF‐κB, JAK/STAT, and MAPK pathways will provide deeper mechanistic insight.

## Funding

This study was funded by the Shiraz University.

## Conflicts of Interest

The authors declare no conflicts of interest.

## Data Availability

The data that support the findings of this study are available upon request from the corresponding author. The data are not publicly available due to privacy or ethical restrictions.
